# The Irrigation Treatment of Gonorrhea

**Published:** 1900-11

**Authors:** 


THE IRRIGATION TREATMENT OF GONORRHEA. Its Local Complications and Sequelæ. By FERD. C. VALENTINE, M. D. Illustrated. New York. William Wood & Company. 1900. [Price, $2.00, net.]

Dr. Valentine states in the brief preface of his "little book" (220 pages and 57 illustrations, some original and some unique,) "that his object was to fill the irrigationless chasm ('hiatus') existing in medical works and patiently wait until abler pens supply the missing link." Hastily glancing through the opening chapter another motive suggests itself. Under the heading "General Considerations" he quotes Goldberg's and other reports to show that 90 per cent. of cures, in fourteen days, is claimed for the irrigation treatment of gonorrhea.

The author also alleges that not a dozen men in the world were using the irrigation treatment in 1894. This statement is certainly rash, as more than that number were using it in Buffalo, and Buffalo is a small part of the world, smaller even than her ambitious citizens thought, previous to the last census. Previous to this time Rona, Janet, Feliké and Guyon, in Europe and others in America had exploited catheterless methods of irrigation of the lower urinary tract.

He states that the so-called "Valentine Method" of treating gonorrhea, is "erroneously misnamed;" that he "did nothing except devise a simple apparatus, develop the technique, modify the medication, render the rules precise and write many articles," which profusely illustrated his device. (Price, $6.50.)

Sure, this was more than nothing, hence an exception was taken. The doctor is diffident. Under the caption, "The Irrigator," there follow six pages of description and cuts, decorated with figures and letters, to make plain the use and manner of use of this simple apparatus. With chain and pulley he hoists his petard 8 or 9 feet high, which gives a hydrostatic pressure of about ½ lb. per square inch for each foot,total pressure of 4 or 5 lbs. To offset this he devises a shut off to control the pressure; not only this, but he also attaches a saucer-dish shield to the nozzle to catch the water forcibly ejected from the urethra. The urethra and bladder resent the impact of forced injections, just as the bowel rejects the forced injection of an enema.

The shield, pulley, chain, elaborate shut-off and controller are expensive, cumbersome and fulfil no useful function. To irrigate the anterior urethra, abundance of irrigant and sufficient force to open its lumen are necessary. For irrigating the urethra in sections the author directs that the penis be grasped so that the distal ends of the fingers press upon the urethral canal in order that by opening one finger after another the wash is done in sections, resembling perhaps the fingering of a flute.

For irrigating the anterior urethra the patient stands or sits upright, holds the glass nozzle in the urethra while abundance of water gently flows into this canal and runs out around the instrument, or through the outflow of a double tube.

To irrigate the deep urethra and bladder the body should be supported in a semi-reclining flexed dorsal position for the purpose of relaxation. The relative position of the neck of the bladder is changed at least half an inch by various postures. Animals hump the back and flex the thighs when emptying the abdomino-pelvic emunctories for the relaxation induced thereby. In this position, with a column of a hot (90° to 120° F.) saline solution dilating the urethra, a rhythmic vermicular, a retroperistaltic action is induced, which sucks or swallows the fluid into the bladder. This result may be delayed on the first trial, and in that case straining, as if to urinate, will bring it about. This knack or control is readily acquired. Force or pressure is not necessary and is often harmful. Forceless irrigation is the treatment for acute cystitis.

On page 158 is shown "Patient in Position during Dilatation," with a Kollman dilator. At first glance it looks like a person swinging in a hammock. The body is slightly flexed at the hips. This position serves no useful purpose, it is neither fish, flesh, nor fowl.

The lithotomic posture with the back raised, thighs flexed and legs supported at right angles with the thighs, relaxes the pelvic supports and attachments of the bladder and gives freedom for manipulation.

Dr. Valentine is aggressive, progressive and well up in the literature of genito-urinary medicine. The title of his book gives an inadequate idea of the many good things contained therein. In spite of its faults it is a valuable work and an addition to the subjects discussed. It should find a place in every medical library.

